# 589. Oral Tablet Vaccination Induces Heightened Cross-Reactive CD8 T Cell Responses to SARS-COV-2 in Humans

**DOI:** 10.1093/ofid/ofab466.787

**Published:** 2021-12-04

**Authors:** Susan Johnson, Clarissa Martinez, Mario Cortese, Josefina Martinez, Shaily Garg, Nadine Peinovich, Emery Dora, Sean Tucker

**Affiliations:** Vaxart Inc., South San Francisco, CA

## Abstract

**Background:**

Covid-19 has accelerated global demand for easily distributed vaccines. Furthermore, as variant SARS-CoV-2 strains that circumvent antibody responses emerge, cross-protective vaccines provide substantial public health benefits. Vaxart is developing a shelf stable oral tablet vaccine that incorporates both the spike (S) and the more conserved nucleocapsid (N) proteins. Vaxart’s vaccine platform uses a non-replicating adenovirus and a TLR3 agonist as an adjuvant.

**Methods:**

In an open-label phase 1 clinical study, 35 healthy subjects received either a single low (1x10^10^ IU; n=15) or high (5x10^10^ IU; n=15) dose of the vaccine candidate VXA-CoV2-1 with a small cohort receiving 2 low doses. PBMCs were taken at pre- and 7 days post-vaccination and restimulated with S and N peptides from SARS-CoV-2 or the 4 human endemic coronaviruses (HCoV). Cells were stained for CD4/CD8/CD107a (surface) and IFNγ/TNFα (intracellular). Subjects that received an intramuscular (i.m.) mRNA vaccine had PBMCs taken at the same timepoints and were compared in the same assay.

**Results:**

The study’s results indicate that the VXA-CoV2-1 tablet was well tolerated. The majority of subjects had an increase in S-specific anti-viral CD8^+^ T cell responses. 19/26 (73%) subjects had a measurable CD8^+^ T cell response on day 8 above baseline, on average 1.5-4.6%. In a comparator experiment with the 2 SARS-CoV-2 i.m. mRNA vaccines, VXA-CoV2-1 outperformed other vaccine candidates with a >3.5-fold increase in S specific antiviral CD8 T cell responses. T cell responses specific to the 4 endemic HCoV were increased by 0.6% in subjects given VXA-CoV2-1.

**Conclusion:**

Here we describe a room temperature stable tablet that induces SARS-CoV-2 S specific CD8 T cells of high magnitude after one dose in humans. Overall, the level of antiviral SARS-CoV-2 specific T cells, particularly IFNg-producing CD8s, induced following oral immunization with VXA-CoV2-1 are of higher magnitude than the mRNA vaccines currently in use against COVID-19. T cell responses against 4 endemic HCoV were also induced. Because T cells may be important in protecting against death and severe infection, these results suggest that VXA-CoV2-1 could be cross-protective against a wide array of emerging pandemic coronaviruses.

**Disclosures:**

**Susan Johnson, PhD**, **Vaxart** (Employee) **Clarissa Martinez, MPH**, **Vaxart** (Employee) **Mario Cortese, PhD**, **Vaxart** (Employee) **Josefina Martinez, n/a**, **Vaxart** (Employee) **Shaily Garg, BS**, **Vaxart** (Employee) **Nadine Peinovich, MPH**, **Vaxart** (Employee) **Emery Dora, n/a**, **Vaxart** (Employee) **Sean Tucker, PhD**, **Vaxart** (Employee)

